# Psychiatric Assistance Dog Use for People Living With Mental Health Disorders

**DOI:** 10.3389/fvets.2019.00166

**Published:** 2019-06-06

**Authors:** Janice Lloyd, Laura Johnston, Julia Lewis

**Affiliations:** ^1^Discipline of Veterinary Sciences, James Cook University, Townsville, QLD, Australia; ^2^Independent Researcher, Sydney, NSW, Australia

**Keywords:** assistance dogs, disability, human-animal bond, human-animal relationships, mental health, psychiatric assistance dogs, service dogs

## Abstract

A psychiatric assistance dog (PAD) is a service dog that is trained to assist its handler (owner) who has been diagnosed with a mental health condition such as post-traumatic stress disorder (PTSD), schizophrenia, depression, anxiety, or bipolar disorder. Literature searches reveal that little is known about the population of people who own PADs, the types of dogs used or the functions they provide. One third (*n* = 199) of PAD owners in Australia registered with the charity “mindDog” participated in an online survey designed to better understand the person and dog team. Participants learned about PADs through the internet (37%), health care practitioners (32%), or family/friends (30%). The dogs in the sample were of varying age, gender and breed. The most common reasons for people to choose a dog to be a PAD were temperament (60%) and size/weight (48%). Just under half (48%) of the dogs had been acquired by the owner specifically to be trained as a PAD, and the rest were existing pets. All the dogs were trained by the owner or a combination of the owner and a qualified trainer; none were trained exclusively by assistance/service dog provider organizations. The median age of the participants at the time of data collection was 47 years, ranging from 10 to 75 years. Most (77%) identified as female. Depression (84%), anxiety (social 61%; generalized 60%), PTSD (62%), and panic attacks (57%) were the most reported mental health diagnoses. Tasks the dogs performed for their owners included: reduction of anxiety through tactile stimulation (94%); nudging/pawing to bring back to the present (71%); interrupting undesirable behavior (51%); constant body contact (50%); deep pressure stimulation (45%) and blocking contact from other people (42%). PAD usage decreased (46%), increased (30%) or did not change (24%) participants' use of psychiatric or other health care services. Decrease in service use was mainly due to reduced suicide attempts, and less requirement for hospitalization and medication; increased use was mainly due to enhanced ability to attend appointments. Results of this study show that PAD owners have differing mental health diagnoses, and their dogs perform different tasks to support them in daily life. Every participant described the relationship with his/her PAD as positive, suggesting that a successful working partnership does not require the dog to have been bred or raised specifically for the role. A better understanding of this population and the person-dog relationship will inform the appropriate choice, training and use of PADs for people living with mental health problems.

## Introduction

Dogs and other animals have been helping people with physical disabilities and providing emotional support for centuries, with the first therapeutic use reported in the ninth century (1). Nowadays, assistance dogs (or service dogs) are trained to perform tasks to mitigate a range of physical, psychiatric, or intellectual disabilities for their handlers (owners) (2) as well as being trained for public access. A psychiatric assistance dog (PAD) is a specific type of service dog that is trained to assist its owner who has been diagnosed with a mental health condition, such as post-traumatic stress disorder (PTSD), schizophrenia, depression, anxiety, or bipolar disorder. In Australia, PADs, like other assistance dogs including guide dogs and hearing dogs, are covered under the Commonwealth Disability Discrimination Act 1992 that guarantees public access for all dogs trained as assistance dogs. PADs are distinct from emotional support dogs (ESDs) (sometimes called therapy dogs). An ESD (or other animal) is a pet that provides emotional support to an individual to relieve various disabling conditions. However, the animal is not necessarily *trained* to do so, and service dog legislation in Australia does not permit an ESD to access public areas where dogs are normally prohibited.

PADs can be of any breed or size suitable for the intended purpose of helping people to access public places, travel on public transport and take part in social activities that are “closed off” to them. PADs can be trained by the person who will become the dog's handler (owner-trainer) or in combination with a qualified trainer, while others are trained exclusively by assistance/service dog provider organizations. In Australia, anyone who has been diagnosed with a mental health condition by a medical doctor or other suitable health care professional is eligible to apply to accredit such a dog. However, literature searches reveal that little is known about the population of people who own PADs inclusive of mental health diagnoses, origins and types of dogs used or the functions they provide. A better understanding of peoples' needs and the relationship between owners and their dogs will help inform the appropriate choice, training and use of assistance dogs for people living with mental health issues. Hence, PAD owners (clients) registered with the charity “mindDog” were invited to participate in an anonymous on-line survey to explore these matters.

**Figure 1:**
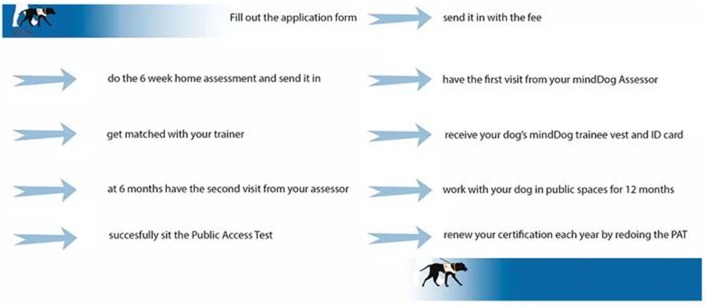
The mindDog accreditation process (www.minddog.org.au/the-process).

mindDog is an Australian not-for-profit organization that helps people who have been diagnosed with a mental health condition/s procure, train and accredit PADs. Information on the mindDog accreditation process can be found in Box 1 (the application form) and Figure 1 (assessment, training and follow-up of the person-dog team). More information on mindDog, including the training standard and the Public Access Test (PAT), can be found at www.minddog.org.au/.

Box 1 **Summary of the mindDog application form.**

The application form for accreditation of a mindDog is in three parts and includes:

Part 1: Details about the applicant and the dog: Ensuring dogs are of an appropriate age, desexed, microchipped, registered, vaccinated, and have access to suitable veterinary care.

Parts 2 & and 3: The opinion of the applicant's health care provider, and other referee, regarding the applicant's ability to care for a dog and how the dog might assist the applicant.

The application form also seeks information on assurance of care for the dog if the owner was unable to do so.

## Materials and Methods

All active clients (*N* = 600) registered with mindDog in February 2018 were invited to participate in an anonymous survey via SurveyMonkey cloud-based software. Questions were forced-choice, multiple-choice, “other” (for free-text to be inserted) or binary (yes/no). Comments on peoples' relationships with their dogs were also sought. Chi-square tests for independence were performed to assess potential associations between owner diagnosis and: the tasks the dog performed, the type of dog used, and the likelihood of changes to health service utilization.

The descriptive results of the survey are presented below. The data obtained from the open-ended (comments) section on peoples' relationships with their dogs was coded into categories and themes, as per Wang and Park [(3), p. 224] process of qualitative coding. While a full thematic analysis is outside the scope of this article, and will be published elsewhere, a synopsis of this preliminary data is presented below.

## Results

### Owner Demographics

One third (*n* = 199; 33%) of eligible people (*N* = 600) completed the survey. The median age of the participants at the time of data collection was 47 years, and age ranged from 10 to 75 years. The majority of the sample (77%) identified as female, and most (58%) lived in suburban areas. Participants learned about PADs through the internet (37%), their health care practitioner (32%), or family/friends (30%).

**Figure 2:**
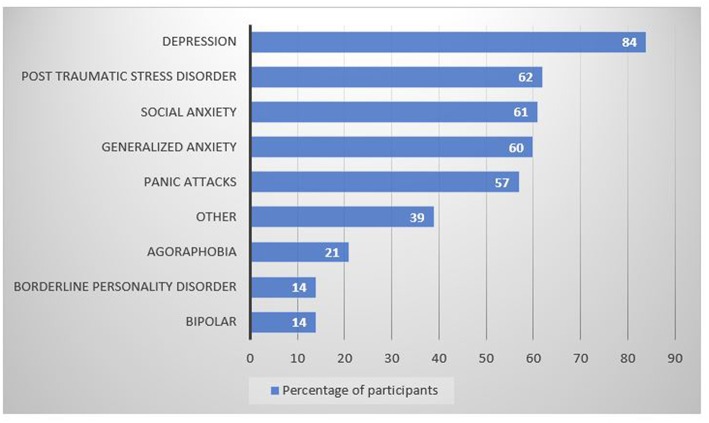
Percentage of participants (*N* = 199) diagnosed with specific mental health conditions.

Depression (84%), anxiety (social 61%; generalized 60%), PTSD (62%) and panic attacks (57%) were the most self-reported mental health diagnoses of this population (Figure 2), with many clients citing multiple diagnoses. Frequently reported mental health diagnoses in the “other” category included Obsessive-Compulsive Disorder, Autism Spectrum Disorder (ASD) and eating disorders.

### Dog Demographics

The breed of dogs in the sample varied widely with several dozen purebred and crossbred breeds identified. Age ranged from around 1- > 10-years; gender was evenly distributed. Most dogs were acquired from a registered breeder (48%) followed by an animal shelter (21%) and non-registered breeders (16%).

The most common reasons for people to choose a dog to be a PAD were temperament (60%) followed by size/weight (48%), with only 15% of participants saying that they chose the dog based on its physical appearance. Just under half (48%) of the dogs had been acquired by the owner specifically to be trained as a PAD, and the rest were existing pets.

All the dogs were trained by either the owner or a combination of the owner and a qualified trainer; none were trained exclusively by assistance/service dog provider organizations.

### Tasks

**Figure 3:**
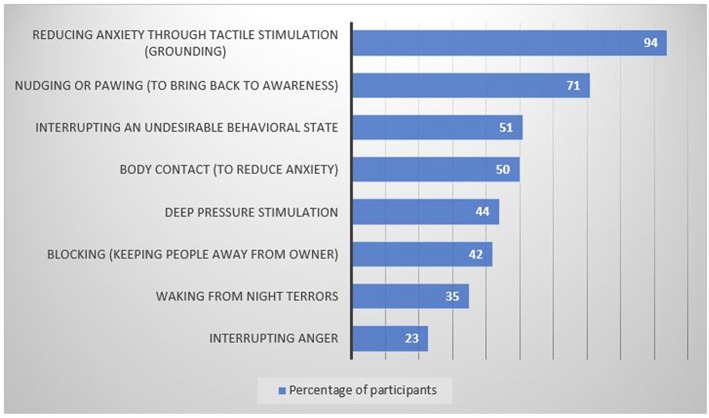
Tasks performed by the psychiatric assistance dogs for the participants (*N* = 199).

All dogs performed multiple tasks for their owners. The most common tasks performed were: reducing anxiety through tactile stimulation (grounding) (94%); nudging or pawing to bring back to the present (71%); interrupting an undesirable behavioral state (51%); constant body contact (50%); deep pressure stimulation (45%); and blocking contact from other people (42%) (Figure 3).

The most common tasks listed in the “other category” were: “making” the owner leave his/her bed/house; “reminding” the owner to take his/her medication; keeping the owner “safe”; “sensing” owner's emotions and behaviors and thus preventing manifestation of an undesirable behavioral state; and providing a “reality check” from anxiety or dissociation/hallucination.

### Outcomes

PAD usage decreased (46%), increased (30%), or did not change (24%) participants' use of psychiatric or other health care services. An analysis of the accompanying narrative pertaining to changes in the use of psychiatric or other health care services revealed that reductions in use of services were mainly due to reduced suicide attempts, less need for hospitalizations, and less requirement for medication. Increased service use was mainly due to enhancement of the owners' ability to attend appointments, as the presence of the dog increased peoples' confidence—both in venturing outdoors and in interacting with others.

No statistically significant associations were found between the owners' mental health diagnoses and: the tasks the dog performed, the type of dog used, and the likelihood of changes to health service utilization. No other relationships within the dataset were found.

### Owner-Dog Relationship

Several themes emerged from the preliminary thematic analysis of the owner-dog relationship including: Independence; Confidence; Social function; Companionship; Safety and Hope. Every pertinent response (*n* = 198) to the question: “What does your mindDog mean to you?” indicated a positive partnership, as exemplified by the following [de-identified] quotes:

“Before I had [my dog] I was so anxious I couldn't even leave the house and I had never had someone to look after before. She has changed my life so much; everyone I know says it and my psychiatrist thinks she's amazing. Once [my dog] became qualified as a minddog I have been able to travel to so many more places and be able to do things independently. I don‘t think I could have done that without her. This also means that I can do things on my own now that in the past I would have needed more help with or been in hospital. But I still definitely need also other health services to help me. She is very good but she can’t replace everyone! But I really hope your research shows how great they are because I don't know how I would cope without her.”

“My assistance dog has allowed me to become more social and allowed me to do some of the most basic life necessities ie: go shopping, leave the house, do university, feel safe when out and about and reduce my anxiety and panic attacks. By having my dog, I have managed to reduce my mental health inpatient stays to just stabilisation admission rather than crisis admission. I can now go out and be active with my children and live a fairly normal life.”

Other data showed that the publics' attitude could be a cause of stress for the owner:

“When I'm with her I don't worry that I'm out, because it's like I have my home with me so it's okay. So I can only say that I am so grateful that psychiatric dogs are now recognised and I hope it only spreads more. That being said, sometimes I find having her with me stressful because sometimes other people start challenging me about having her, even though I have all her certification and ID and vest, and that's really stressful for me when people pay attention to me in such a negative way. So I hope it becomes more widely accepted and less criticised by other people who don't really understand.”

## Discussion

The results of the present study indicate that PADs assist people of all ages, including children, with a range of mental health problems, whose lives are often severely compromised by anxiety and fear, to access public places, travel on public transport and take part in social activities that may have been closed off to them. Although the study was a self-report measure and therefore limited by selection-bias and subjectivity, every relevant comment (*n* = 198) regarding the meaning of the person-dog relationship (i.e., response to the question: “What does your mindDog mean to you?”) was positive. Thus, suggesting that sound conclusions can be drawn about their efficacy.

A plethora of dog breeds were used by the participants in this study—from the Chihuahua to the Irish Wolfhound, illustrating that a PAD does not need to be a certain size or breed (or gender). Indeed, only 15% of participants chose a dog based on its physical appearance. Because PADs come in many shapes and sizes, they can look different to other assistance/service dogs such as the Labrador or Golden retriever commonly used as guide dogs (4). As indicated in the present study, this can lead to stress-provoking attention from the public, as unlike some people who are blind or vision-impaired or have mobility issues, there may be no outward sign of disability. Mental illness frequently carries a heavy social (and self-) stigma (5), and the owner may be reluctant to explain the dog's role. Public education regarding the expanding roles of contemporary service dogs and associated etiquette would help to alleviate social issues with accessibility.

It is noteworthy that over a fifth (21%) of dogs in the study were acquired from an animal shelter suggesting that “rescue” dogs can be an important source of successful PADs. Sourcing dogs from animal rescues or shelters is beneficial in reducing the number of animals killed due to overcrowding and opens up shelter space for another animal who might desperately need it.

The authors hypothesized that there might be an association between the owners' mental health diagnoses and the tasks the dogs performed, but no relationship was found. This is likely due to the variables “diagnosis” and “tasks” being highly confounded as, for example, the majority of people (84%) identified as being diagnosed with depression, and almost all (94%) dogs performed the task of “grounding” for their owners. Future research with only open-ended questions for these variables, rather than forced-choice options as per the present study, which can lead participants to make certain choices, would be valuable. While it is not yet understood what cues, whether behavioral, olfactory, or other, PADs may be responding to when performing tasks, it is clear that the relationship between individual owners and his/her dog is a personal one, influenced by each owner's diagnosis and needs.

As part of the mindDog application process (Box 1), the applicant's health care practitioner completes a form that expresses how the practitioner expects a mindDog might assist the applicant. However, some health care practitioners may not be aware of the roles the dogs can provide, and it is likely that the functions are greater and more varied than are those predicted. Findings from the present study supports the view of the Psychiatric Service Dog Society (PSDS) in the US (6) that PADS be used as an adjunct to ongoing standard-of-care mental health treatments, and not as a substitution. These findings can be used to inform medical doctors and other health care providers, who play a pivotal role in their patients' application process for a “mindDog,” about how the dogs may be of assistance.

A review on the effectiveness of a range of assistance animals (AA) for Australia's National Disability Insurance Agency (NDIA) (7) concluded that there may be large economic benefits to AA ownership, including the ability to work, attend school and concerning services no longer required (e.g., a non-verbal child with ASD who now speaks). Although evidence is limited, the results of the present study support this conclusion in that nearly half (46%) of participants said that their use of psychiatric and other health services had decreased—mainly due to reduced suicide attempts, and less requirement for hospitalization and medications. Public hospital spending in Australia has been the single fastest growing area of government spending over the past decade or so (8). From a health economic perspective, judicious decreased use of services and hospitalizations/use of medications is likely to save money.

Howell et al. (7) also recommended that should AAs be provided by the NDIA, the standard for assistance dog training (inclusive of PADs) should adopt the model of the AA provider organization selecting/breeding and training dogs for AA roles—a process that typically takes around 2 years. However, the findings of the present study suggests that successful working partnerships does not require the PAD to have been bred and/or raised specifically for the role, as every participant considered their personal and working relationship with their dog to be effective despite no dogs being acquired/trained by this method. The so-called “human-animal bond” is the dynamic relationship between people and animals that influences the psychological and physiological states essential to the health and well-being of both (9). Unlike many service dog organizations, mindDog works with existing pets so a strong owner-dog bond is likely to be already in place. Thus, it is the authors' opinion that while many assistance dogs (such as guide dogs, hearing dogs and others trained to assist individuals and their families impacted by disability) be exclusively acquired and trained by AA provider organizations, this approach may not be necessary for PADs. This could have far-reaching consequences for people who wish to use such a dog as waiting times and financial costs for a trained dog could be dramatically reduced.

There appears to be a growing need for PADs to help individuals with psychiatric disabilities. A recent study by Walther et al. (10) showed that PADs placed fourth in North American accredited placements of various assistance dogs, surpassing the number of hearing dogs placed. Indeed, the number of applicants to mindDog has doubled at the time of writing this article (9-months since gathering the data), resulting in the organization having to limit when it can accept applications. When thinking about the direction the field may take in the future it seems unlikely that PAD activities are likely to end, therefore steps must be taken to ensure the well-being of the dogs as well as the handler in this remarkable example of the human-animal bond in action. Responsible pet ownership requires a commitment to provide for all the requirements of one's pet—food, exercise, housing, reward-based training, love and affection, grooming, and veterinary care. While mindDogs only works with positive force-free training methods [as recommended by (11)], it is imperative for all owners to understand how animals communicate and learn, and to thoroughly research the basics of pet care before acquiring any new pet to ensure she/he has the capacity to meet the physiological, behavioral and social needs of the animal. Future research should focus on Shubert's (2) advice whereby handlers (and trainers) become adept in canine body language, recognize signs of stress in dogs, have realistic expectations, and ensure only dogs with the appropriate temperament be trained as PADs.

## Conclusion

This study has contributed to the small but growing body of research on PADs including the demographics of people who use these dogs in Australia, the origin and type of dogs used and the functions the dogs provide. PADs can be all shapes and sizes and perform a plethora of roles that provide substantial benefits to a broad range of people. In addition to training, it appears that for a satisfactory relationship, PADs do not require to have been bred or raised specifically for the role, but that success hinges on the human-animal bond. An understanding of the relationship between owners and their dogs will help inform the appropriate choice of dog, training and use of assistance dogs for people living with mental health issues to better support the needs of both species.

## Ethics Statement

The study was carried out in accordance with the recommendations of James Cook University Human Ethics Committee *(*Ethics Approval Number H7210) with informed consent from all subjects. The participants in the study were clients of mindDogs, and had been diagnosed with a mental health condition by a qualified health professional.

## Author Contributions

JaL, LJ, and JuL contributed to the design, delivery and analyses of this work. JaL wrote the article with the approval of LJ and JuL, who have critically revised the content. JaL, LJ, and JuL agree to be accountable for the content.

### Conflict of Interest Statement

LJ is a board member of the charity mindDog. The remaining authors declare that the research was conducted in the absence of any commercial or financial relationships that could be construed as a potential conflict of interest.
